# Integrated Hospital, Emergency Department, and Community Surveillance for Respiratory Viruses in Milan, Italy

**DOI:** 10.1002/jmv.71073

**Published:** 2026-07-21

**Authors:** Alberto Rizzo, Federica Salari, Cristina Galli, Manuel Maffeo, Cristina Paduraru, Simone Villa, Alberto Dolci, Elena Pariani, Danilo Cereda, for the Milan Respiratory Viruses Surveillance Working Group, Andrea Cavallo, Sara Giubileo, Alessandra Lombardi, Davide Mileto, Loriana Morelli, Stefano Odelli, Sandro Binda, Emanuela Matteucci, Valeria Primache, Arlinda Seiti, Gabriele del Castillo, Giovanni Rezza, Carlo Signorelli

**Affiliations:** ^1^ Laboratory of Clinical Microbiology, Virology and Bioemergencies Luigi Sacco University Hospital, ASST Fatebenefratelli Sacco, Regional Center for Infectious Diseases (CEREMI), Lombardy Region Milan Italy; ^2^ Department of Biomedical Sciences for Health University of Milan Milan Italy; ^3^ Directorate General for Health Lombardy Region Milan Italy; ^4^ Department of Biomedical and Clinical Sciences University of Milan Milan Italy; ^5^ Medical Directorate Luigi Sacco University Hospital, ASST Fatebenefratelli Sacco Milan Italy; ^6^ National PhD Programme in One Health Approaches to Infectious Diseases and Life Science Research, Department of Public Health, Experimental and Forensic Medicine University of Pavia Pavia Italy; ^7^ School of Medicine University Vita‐Salute San Raffaele Milan Italy

**Keywords:** influenza virus, public health, respiratory virus, RSV, SARS‐CoV‐2, surveillance

## Abstract

Respiratory virus circulation may vary across healthcare settings and age groups, potentially limiting the interpretability of surveillance based on a single source. In Milan, Italy, established community‐ and emergency department‐based systems coexist with hospital laboratory testing, but hospital ward‐level data are not routinely integrated. The aims of this study were to describe respiratory viruses' epidemiology and assess whether ward‐based surveillance complements community and ED surveillance. We analyzed molecular test results from respiratory samples collected from January 1, 2024 (W1/2024) to October 12, 2025 (W41/2025) in community, ED, and hospital ward settings. Viruses included influenza A (IAV), influenza B (IBV), RSV, and SARS‐CoV‐2 (with extended panels in a subset). Among 8029 samples, overall positivity for ≥ 1 virus was 46.8%, highest in ED (51.1%) and community (50.5%) and lower in wards (36.7%) (*p* < 0.001). During 2024–2025, IAV was first detected in wards (W33/2024) and peaked earlier in wards (W2/2025) than community (W3/2025) and ED (W4/2025). IBV was first detected in ED (W36/2024) and peaked in ED (W5/2025), community (W7/2025), and wards (W8/2025). RSV reappeared first in community (W37/2024) and peaked in community/ED (W50/2024) and later in wards (W2/2025). Respiratory virus positivity and descriptive peak timing differed across healthcare settings and age groups. Ward‐based surveillance provided complementary information for hospitalized and older populations, but it was not consistently earlier or superior to community or ED surveillance. Integrated, multi‐setting surveillance can improve situational awareness when interpreted alongside differences in tested populations, testing indications, and clinical outcome availability.

## 1 Background

The circulation patterns of respiratory viruses may differ depending on the season, healthcare setting, and age group. A study conducted in the Netherlands has shown that severe acute respiratory infections (SARI) in intensive care units (ICUs) often increased earlier than medically attended influenza cases in primary care, suggesting a potential early warning system in secondary care. This study has highlighted the importance of hospital surveillance in providing valuable insights into vulnerable populations [1]. However, a recent study from France has reported that community‐based surveillance captured earlier and broader circulation of influenza virus and severe acute respiratory syndrome coronavirus 2 (SARS‐CoV‐2) in young adults, who are less likely to be hospitalized [2]. Furthermore, significant differences have also been observed in the distribution of viruses by setting (community vs. hospital) and by age group, corroborating existing evidence of setting‐ and age‐specific severity patterns of certain respiratory viruses [3]. Taken together, these heterogeneous findings support the need for national and local, virus‐specific analyses based on laboratory confirmation through both community‐ and hospital‐based surveillance programs.

In Italy, the Ministry of Health has established RespiVirNet, an integrated sentinel surveillance system of respiratory infections based on primary general practitioners (GPs) and pediatricians, covering around 4% of the Italian population, and providing information on influenza and other respiratory viruses circulation [4]. At a regional level, a syndromic surveillance system based on emergency department (EDSyS) was implemented across the Lombardy region (population ≈ 10 million), one of the most populous regions in the European Union (EU), to provide early warnings of public health issues such as respiratory viruses [5].

We hypothesized that the timing of respiratory virus circulation differs across healthcare settings. Specifically, we investigated whether laboratory detections in hospital wards precede or follow detections in the ED and community, depending on the virus and the age groups involved. Accordingly, this study aimed to assess whether incorporating a hospital ward‐based surveillance component into current surveillance systems could provide complementary information on respiratory virus circulation across care settings.

The aim of the study was to describe the epidemiology of respiratory virus circulation in Milan, and to assess whether hospital ward‐based surveillance could complement existing community‐ and ED‐based surveillance programs.

## 2 Methods

### 2.1 Data Sources and Variables

The study included respiratory samples collected from January 1, 2024 (ISO week W1/2024) to October 12, 2025 (W41/2025) from individuals with respiratory infections by: (a) sentinel GPs and pediatricians participating in the RespiVirNet surveillance program in the ATS Metropolitan Milan area, a primary public health agency covering approximately 3.5 million inhabitants, (b) physicians in four EDs, and (c) physicians in general wards and ICUs of the ASST Fatebenefratelli Sacco.

The ASST Fatebenefratelli Sacco is a health and social care organization comprising four hospitals in Milan. It has a total capacity of 900 beds, and its 4 EDs reported approximately 179 000 visits in both 2023 and 2024.

The inclusion criteria were: (a) presenting with an acute respiratory syndrome consistent with influenza‐like illness (ILI) or acute respiratory infection (ARI) during the study period, (b) age ≥ 1 year, and (c) providing a respiratory sample [nasopharyngeal swab (NPS) or a bronchoalveolar lavage (BAL)]. Infants under 1 year were excluded because they represent a clinically distinct group with different hospitalization thresholds and testing practices.

Operationally, ILI was defined according to the surveillance protocols as sudden symptom onset with at least one systemic symptom (fever or feverishness, malaise, headache, or myalgia) and at least one respiratory symptom (cough, sore throat, or shortness of breath) [6, 7]. ARI was defined as acute onset of at least one respiratory symptom, such as cough, sore throat, shortness of breath, or coryza, judged clinically compatible with an acute infectious respiratory syndrome [6, 7].

The data set included age group, biological sex, specimen collection date, virus target, and the qualitative result (positive/negative) of molecular tests. The age groups included were: 1–5, 6–15, 16–25, 26–45, 46–65, and > 65 years [8].

All data were pseudonymized and stored on secure institutional servers for regional and national surveillance programs [4, 5].

### 2.2 Institutional Surveillance Programs for Respiratory Virus Surveillance

RespiVirNet is the Italian national integrated system for influenza and other respiratory viruses' surveillance [4]. The Lombardy EDSyS complements this system by systematically sampling a fixed number of patients with acute respiratory symptoms in participating EDs [5].

The Laboratory of Clinical Microbiology, Virology and Bioemergencies (CLIMVIB) at the ASST Fatebenefratelli Sacco and the Public Health Laboratory (PHL) at the Department of Biomedical Sciences for Health, University of Milan, function as designated reference laboratories within both programs.

### 2.3 Sample Collection and Laboratory Testing

In the community setting, NPS were collected by sentinel GPs and pediatricians and tested at the PHL. All samples were tested using Clonit FluA/FluB/SARS‐CoV‐2 (Clonit s.r.l., Italy) and the Viasure Respiratory Panel III Real Time PCR Detection Kit (Viasure – Certest Biotec S.L., Spain) to detect influenza A virus (IAV), influenza B virus (IBV), respiratory syncytial virus (RSV), parainfluenza viruses 1–4 (PIV), human adenovirus (AdV), human coronavirus (hCoV), 229E, NL63, OC43, HKU1, and SARS‐CoV‐2.

In the hospital, including both EDs and wards, NPS and BAL were processed at the CLIMVIB and tested by using real‐time molecular assays Xpert Xpress CoV‐2/Flu/RSV plus (Cepheid, California, USA) or Allplex Respiratory Panel 1 (Seegene Inc., Republic of Korea), Allplex Respiratory Panels 2 and 3 (Seegene Inc., Republic of Korea) to detect IAV, IBV, RSV, PIV, AdV, hCoV, and SARS‐CoV‐2.

In the ED setting, patients were sampled within the Lombardy EDSyS framework among those presenting with acute respiratory symptoms, according to the regional surveillance protocol. Ward‐based testing was performed as part of routine clinical care at ASST Fatebenefratelli Sacco. In general wards and ICUs, testing was requested by treating physicians for hospitalized patients with suspected viral respiratory infection, including acute respiratory symptoms, pneumonia, or clinical deterioration in which a respiratory virus diagnosis was considered relevant for patient management or infection prevention. Therefore, in contrast to sentinel community surveillance and the ED syndromic surveillance framework, ward testing was not based on a fixed sampling denominator and may have been influenced by clinical severity, comorbidity, and local testing practices.

Both laboratories participate in institutional external quality assessment programs, ensuring harmonized and reliable results.

**Figure 1:**
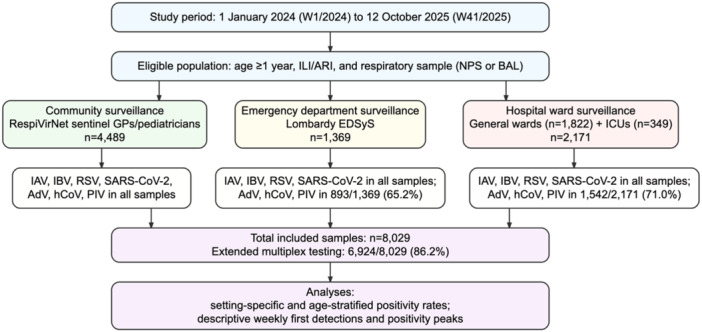
Study flowchart. The flowchart summarizes the surveillance settings, inclusion framework, sample numbers (*n*), and common (IAV, IBV, RSV, SARS‐CoV‐2) versus extended (common + AdV, hCoV, PIV) molecular testing panels. AdV = adenovirus; ARI = acute respiratory infection; BAL = bronchoalveolar lavage; ED = emergency department; EDSyS = emergency department syndromic surveillance system; GP = general practitioner; hCoV = seasonal human coronavirus; IAV = influenza A virus; IBV = influenza B virus; ICUs = intensive care units; ILI = influenza‐like illness; NPS = nasopharyngeal swab; PIV = parainfluenza virus; RSV = respiratory syncytial virus; SARS‐CoV‐2 = severe acute respiratory syndrome coronavirus 2.

A schematic overview of study inclusion, care settings, sample types, and laboratory testing pathways is provided in Figure 1.

### 2.4 Statistical Analysis

The primary descriptive outcomes were virus‐specific positivity rates by setting, week, and age group. Weekly positivity rates were calculated by dividing the number of positive samples by the number of samples tested for the corresponding virus in the corresponding week. Positivity for one or more viruses was calculated as the proportion of samples positive for at least one virus among the targets tested for that sample; samples with co‐detections were counted once in this overall outcome.

For the 2024–2025 Autumn/Winter season, defined as September 1, 2024 (W35/2024) to February 28, 2025 (W9/2025), we described the timing of first detections and the week of the highest weekly positivity for the viruses with the clearest seasonal circulation during this period, namely IAV, IBV, and RSV [9].

Weekly case counts were inspected alongside weekly positivity rates to avoid overinterpreting isolated high positivity values when denominators were small. First‐detection and peak‐week comparisons were treated as descriptive indicators of temporal patterns, not as formal statistical tests of lead–lag relationships between settings.

Aggregate characteristics and positivity rates across community, ED, and ward settings were compared using Fisher's exact test or chi‐square (*χ* ^2^) test, as appropriate. Statistical significance was set at *p* < 0.05. The statistical analysis was performed using RStudio software version 2026.01.0 + 392 [10].

## 3 Results

### 3.1 Study Population, Testing Panels, and Overall Positivity

This study included 8029 respiratory samples from as many ILI/ARI cases: 4489 (55.9%) were collected by sentinel GPs/pediatricians in the ATS metropolitan Milan area, 1369 (17.1%) from patients attending the 4 EDs, and 2171 (27.0%) were collected from patients hospitalized in general wards (*n* = 1822) or ICUs (*n* = 349) of the ASST Fatebenefratelli Sacco.

All samples were tested for IAV, IBV, RSV, and SARS‐CoV‐2. Most (6924/8029, 86.2%) samples underwent extended testing for IAV, IBV, RSV, PIV, AdV, hCoV, and SARS‐CoV‐2. Extended testing included all community samples, 65.2% (893/1369) of ED samples, 74.0% (1349/1822) of general ward samples, and 55.3% (193/349) of ICU samples.

The overall positivity rate for one or more viruses among the targets tested for each sample was 46.8% (3761/8029), with the highest rates observed in the ED (51.1%; 699/1369) and community (50.5%; 2266/4489), followed by the ward setting (36.7%; 796/2171) (*p* < 0.001). General wards and ICUs were analyzed together because no significant differences in overall positivity were observed between them (*p* > 0.05).

### 3.2 Setting‐Specific Virus Positivity

The overall positivity rate by virus was 7.5% (602/8029) for IAV, 5.5% (442/8029) for SARS‐CoV‐2, 5.4% (373/6924) for AdV, 5.2% (419/8029) for IBV, 5.1% (351/6924) for PIV, 4.2% (340/8029) for RSV, and 3.3% (228/6924) for hCoV. Denominators for AdV, hCoV, and PIV were smaller because extended testing was performed in all community samples but only in a subset of ED and ward samples.

The two most frequently detected viruses were IAV and IBV in the community setting (8.4%, 378/4489, and 5.8%, 262/4489, respectively) and in the ED setting (10.7%, 147/1369, and 9.3%, 127/1369, respectively), whereas PIV (4.7%, 72/1542) and SARS‐CoV‐2 (4.4%, 96/2171) were the most frequently detected viruses in the ward setting. Positivity differed significantly across the three settings for all viruses except hCoV (Table 1).

**Table 1:** Population characteristics and virus positivity by setting.

	Community setting		ED setting		Ward setting		
	(*N* = 4489)	%	(*N* = 1369)	%	(*N* = 2171)	%	*p*
*Biological sex*	*n*		*n*		*n*		
Female	2459	54.8	616	45.0	1032	47.5	< 0.001
Male	2030	45.2	753	55.0	1139	52.5	
*Age groups*							
1–5	1230	27.4	426	31.1	465	21.4	< 0.001
6–15	877	19.5	238	17.4	191	8.8	< 0.001
16–25	311	6.9	44	3.2	41	1.9	< 0.001
26–45	979	21.8	85	6.2	99	4.6	< 0.001
46–65	760	16.9	148	10.8	364	16.7	< 0.001
> 65	332	7.5	428	31.3	1011	46.6	< 0.001
*Positivity rate*	*n*/*N*		*n*/*N*		*n*/*N*		
Overall	2266/4489	50.5	699/1369	51.1	796/2171	36.7	< 0.001
IAV	378/4489	8.4	147/1369	10.7	77/2171	3.5	< 0.001
IBV	262/4489	5.8	127/1369	9.3	30/2171	1.4	< 0.001
AdV	252/4489	5.6	59/893	6.6	62/1542	4.0	0.012
hCoV	137/4489	3.1	41/893	4.6	50/1542	3.2	0.062
PIV	257/4489	5.7	22/893	2.5	72/1542	4.7	< 0.001
RSV	181/4489	4.0	87/1369	6.4	72/2171	3.3	< 0.001
SARS‐CoV‐2	258/4489	5.7	88/1369	6.4	96/2171	4.4	0.021

*Note:* Characteristics include biological sex and age groups of individuals with suspected viral respiratory infection tested in the community (general practitioners/pediatricians), emergency department (ED), and ward (hospital general wards plus intensive care units) setting. Laboratory testing included influenza A (IAV) and B (IBV) viruses, adenovirus (AdV), seasonal human coronaviruses (hCoV), parainfluenza virus types 1–4 (PIV1–4), respiratory syncytial virus (RSV), severe acute respiratory syndrome coronavirus 2 (SARS‐CoV‐2). Denominators for AdV, hCoV, and PIV differ because extended syndromic panels were performed in all community samples and in subsets of ED (893/1369) and ward (1542/2171) cases.

### 3.3 Descriptive Temporal Patterns of IAV, IBV, RSV, and SARS‐CoV‐2

**Figure 2:**
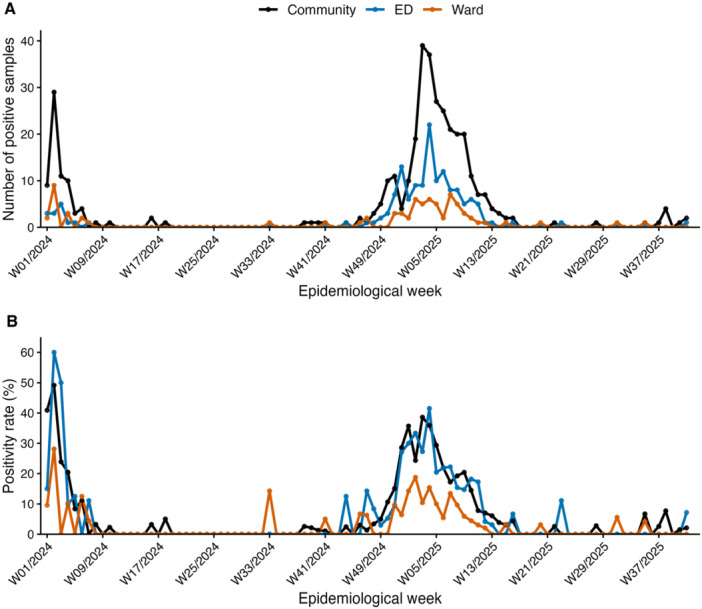
Weekly influenza A virus (IAV) detections and positivity among individuals tested in the community (general practitioners/pediatricians), emergency department (ED), and hospital wards. (A) *X*‐axis measures the epidemiological week (from January 1, 2024, W1/2024, to October 12, 2025, W41/2025); *Y*‐axis measures the number of positive individuals. (B) *X*‐axis measures the week; *Y*‐axis measures the positivity rate (no. of positive cases/no. of tested individuals). Lines show observed weekly values and are intended for descriptive comparison of temporal patterns.

After a low‐activity period in mid‐2024, IAV was detected sporadically first in the ward setting (W33/2024), then in the community (W38/2024), and finally in the ED (W44/2024). The IAV positivity rate peaked in the ward setting in W2/2025 (6/32, 18.8%), approximately 1 week before the community peak (W3/2025; 39/101, 38.6%) and 2 weeks before the ED peak (W4/2025; 22/53, 41.5%). IAV positivity remained above 10% from W50/2024 to W10/2025 in the community, from W52/2024 to W11/2025 in the ED, and from W1/2025 to W7/2025 in the ward setting (Figure 2).

Following the absence of IBV cases during the July–August 2024 period, IBV was first detected in the ED in W36/2024, subsequently in the community in W43/2024, and in wards in W49/2024. The highest positivity rates were observed in W5/2025 in the ED (20/49, 40.8%), W7/2025 in the community (41/122, 33.6%), and W8/2025 in the ward setting (5/52, 9.6%).

**Figure 3:**
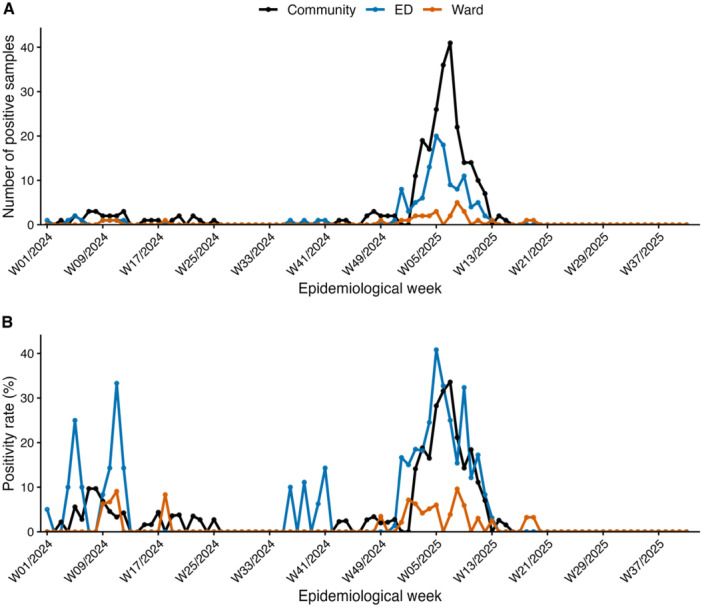
Weekly influenza B virus (IBV) detections and positivity among individuals tested in the community (general practitioners/pediatricians), emergency department (ED), and hospital wards. (A) *X*‐axis measures the epidemiological week (from January 1, 2024, W1/2024, to October 12, 2025, W41/2025); *Y*‐axis measures the number of positive individuals. (B) *X*‐axis measures the week; *Y*‐axis measures the positivity rate (no. of positive cases/no. of tested individuals). Lines show observed weekly values and are intended for descriptive comparison of temporal patterns.

IBV positivity remained above 10% from W52/2024 to W11/2025 in the community and from W2/2025 to W11/2025 in the ED, whereas ward positivity remained below 10% throughout the period (Figure 3).

**Figure 4:**
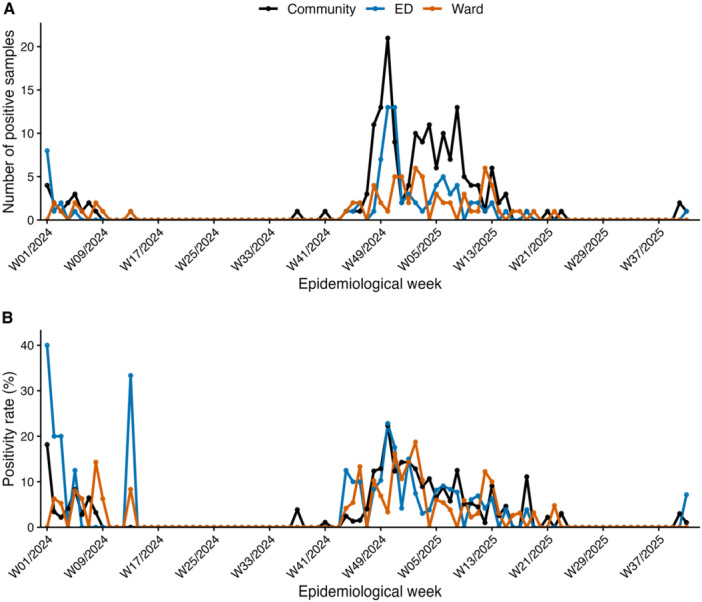
Weekly respiratory syncytial virus (RSV) detections and positivity among individuals tested in the community (general practitioners/pediatricians), the emergency department (ED), and hospital wards. (A) *X*‐axis measures the epidemiological week (from January 1, 2024, W1/2024, to October 12, 2025, W41/2025); *Y*‐axis measures the number of positive individuals. (B) *X*‐axis measures the week; *Y*‐axis measures the positivity rate (no. of positive cases/no. of tested individuals). Lines show observed weekly values and are intended for descriptive comparison of temporal patterns.

RSV demonstrated a seasonal pattern during the study, with two periods of increased activity during early 2024 and late 2024. RSV reappeared first in the community setting in W37/2024, and then in the ED and the ward settings from W44/2024. RSV positivity rates peaked in W50/2024 in the community (22.3%, 21/94) and the ED (22.8%, 13/57) and later, in W2/2025, in the wards (18.8%, 6/32). RSV positivity remained above 10% from W48/2024 to W2/2025 in the community, from W49/2024 to W51/2024 in the ED, and from W51/2024 to W3/2025 in the ward setting (Figure 4).

**Figure 5:**
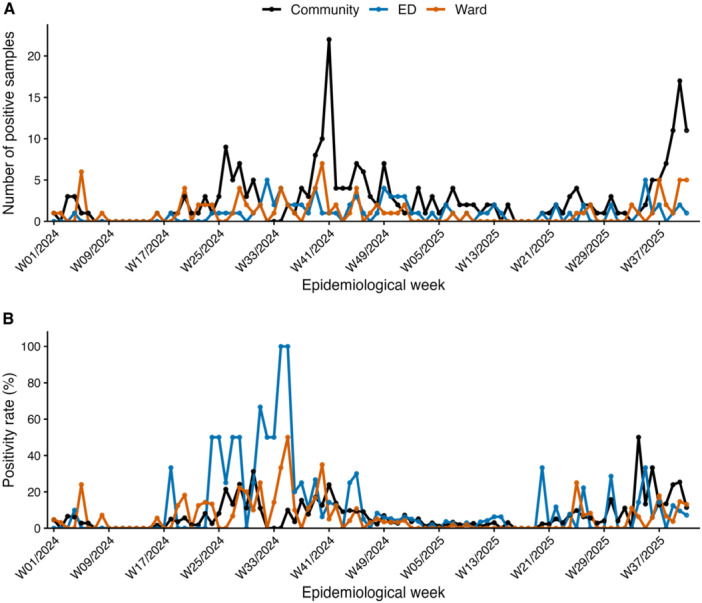
Weekly severe acute respiratory syndrome coronavirus 2 (SARS‐CoV‐2) detections and positivity among individuals tested in the community (general practitioners/pediatricians), emergency department (ED), and hospital wards. (A) *X*‐axis measures the epidemiological week (from January 1, 2024, W1/2024, to October 12, 2025, W41/2025); *Y*‐axis measures the number of positive individuals. (B) *X*‐axis measures the week; *Y*‐axis measures the positivity rate (no. of positive cases/no. of tested individuals). Lines show observed weekly values and are intended for descriptive comparison of temporal patterns.

SARS‐CoV‐2 was detected throughout most of the study period, with higher weekly positivity rate in mid‐2024 (Spring/Summer) and lower‐level circulation during the Autumn/Winter periods. From W24/2024 to W36/2024, increased SARS‐CoV‐2 activity was recorded in all three settings. During the subsequent Autumn/Winter period, positivity decreased but SARS‐CoV‐2 remained detectable in all settings, increasing again approximately from mid‐2025 (Figure 5).

### 3.4 Adenovirus, Seasonal Human Coronaviruses, and Parainfluenza Viruses

AdV, hCoV, and PIV circulated at generally lower levels than influenza and RSV and showed more diffuse temporal patterns. AdV was detected in all settings across most weeks of 2024–2025, with frequent low‐level circulation in the community and intermittent weeks with positivity rates above 10% in both the community and the ED setting, scattered across Spring, Summer, and Autumn.

HCoV activity was limited, with sporadic detections throughout the period and only a few weeks with positivity rates above 10%, mainly in early 2024 and late Summer 2025 in the community setting, occasional higher percentages in the ED, and a single week with ≥ 10% positivity in the ward setting.

PIV circulation was predominantly community‐ and ward‐based, with positivity rates above 10% mainly in Spring 2024, accompanied by later and less frequent ED involvement from Autumn 2024. New increases in positivity rate were observed during Spring/Summer 2025 first in wards, then in the community and later in the ED (Figure S1).

### 3.5 Age‐Stratified Positivity

When stratified by age group, differences in virus positivity rates across settings were most evident in the youngest age group and in adults ≥ 46 years.

Among children aged 1–5 years, IAV and IBV exhibited significant setting‐related variations, with the highest positivity rates observed in the ED setting: 14.6% (62/426) for IAV and 11.5% (49/426) for IBV. In this age group, RSV positivity rate was also highest in the ED (13.6%, 58/426), whereas hCoV was the most frequently detected in the ward setting (7.9%, 25/317). In contrast, PIV showed the highest positivity rate in the community setting (12.9%, 159/1,230).

Among children aged 6–15 years, IBV and AdV exhibited the highest positivity rates in the ED, at 24.4% (58/238) and 9.0% (14/156), respectively. Conversely, hCoV exhibited a slightly higher positivity rate in the ward setting (3.2%, 5/156).

Among younger adults (aged 16–25 and 26–45 years), most viruses showed no statistically significant differences related to setting: the only exception was SARS‐CoV‐2 in the 26–45‐year age group, which was significantly higher in the community setting (8.7%, 85/979).

Among adults aged 46–65 years, IAV, IBV, and SARS‐CoV‐2 had the highest positivity rates in the community: 11.7% (89/760), 4.3% (33/760), and 11.6% (88/760), respectively.

In individuals aged > 65 years, IAV and SARS‐CoV‐2 again had the highest positivity in the ED, at 9.6% (41/428) and 14.3% (61/428), respectively. PIV positivity rates also differed significantly, with the highest positivity rates in the community, 5.1% (17/332). Table 2 summarizes the detection of respiratory viruses across age groups.

**Table 2:** Positivity rates by virus and age group.

	Community setting	%	ED setting	%	Ward setting	%	*p*	Community setting	%	ED setting	%	Ward setting	%	*p*
	1–5 years							6–15 years						
IAV	98/1230	8.0	62/426	14.6	17/465	3.7	< 0.001	66/877	7.5	24/238	10.1	9/191	4.7	0.112
IBV	31/1230	2.5	49/426	11.5	6/465	1.3	< 0.001	75/877	8.6	58/238	24.4	20/191	10.5	< 0.001
AdV	180/1230	14.6	44/291	15.1	54/317	17.0	0.567	45/877	5.1	14/156	9.0	2/156	1.3	0.008
hCoV	42/1230	3.4	22/291	7.6	25/317	7.9	< 0.001	8/877	0.9	4/156	2.6	5/156	3.2	0.037
PIV	159/1230	12.9	13/291	4.5	39/317	12.3	< 0.001	26/877	3.0	5/156	3.2	5/156	3.2	0.977
RSV	97/1230	7.9	58/426	13.6	39/465	8.4	< 0.001	19/877	2.2	8/238	3.4	6/191	3.1	0.489
SARS‐CoV‐2	23/1230	1.9	5/426	1.2	6/465	1.3	0.511	15/877	1.7	2/238	0.8	2/191	1.0	0.535
	16–25 years							26–45 years						
IAV	19/311	6.1	6/44	13.6	1/41	2.4	0.089	83/979	8.5	6/85	7.1	3/99	3.0	0.153
IBV	33/311	10.6	7/44	15.9	1/41	2.4	0.119	87/979	8.9	6/85	7.1	0	0.0	0.567
AdV	5/311	1.6	0	0.0	1/38	2.6	0.646	14/979	1.4	1/62	1.6	1/65	1.5	0.991
hCoV	9/311	2.9	0	0.0	2/38	5.3	0.430	35/979	3.6	1/62	1.6	1/65	1.5	0.498
PIV	6/311	1.9	0	0.0	2/38	5.3	0.194	21/979	2.1	1/62	1.6	1/65	1.5	0.913
RSV	10/311	3.2	0	0.0	1/41	2.4	0.788	22/979	2.2	3/85	3.5	1/99	1.0	0.513
SARS‐CoV‐2	18/311	5.8	2/44	4.5	1/41	2.4	0.648	85/979	8.7	1/85	1.2	1/99	1.0	0.001
	46–65 years							> 65 years						
IAV	89/760	11.7	8/148	5.4	13/364	3.6	< 0.001	23/332	6.9	41/428	9.6	34/1011	3.4	< 0.001
IBV	33/760	4.3	4/148	2.7	1/364	0.3	< 0.001	3/332	0.9	3/428	0.7	2/1011	0.2	0.169
AdV	6/760	0.8	0	0.0	1/225	0.4	0.588	2/332	0.6	0	0.0	3/588	0.5	0.855
hCoV	31/760	4.1	4/103	3.9	4/225	1.8	0.26	12/332	3.6	10/253	4.0	13/588	2.2	0.287
PIV	28/760	3.7	1/103	1.0	8/225	3.6	0.358	17/332	5.1	2/253	0.8	17/588	2.9	0.010
RSV	23/760	3.0	4/148	2.7	6/364	1.6	0.395	10/332	3.0	14/428	3.3	19/1011	1.9	0.217
SARS‐CoV‐2	88/760	11.6	17/148	11.5	21/364	5.8	0.007	29/332	8.7	61/428	14.3	65/1011	6.4	< 0.001

*Note:* Positivity rates (%) of influenza A (IAV) and B (IBV) viruses, adenovirus (AdV), seasonal human coronaviruses (hCoV), parainfluenza virus (PIV), respiratory syncytial virus (RSV), severe acute respiratory syndrome coronavirus 2 (SARS‐CoV‐2), among individuals tested in the community, emergency department (ED), and hospital ward settings. Denominators differ for AdV, hCoV, and PIV because extended syndromic panels were performed in all community samples and in a subset of ED and ward samples. For each age group and virus, *p* values represent the overall comparison of positivity rates across the three care settings (community, ED, and ward settings), using *χ* ^2^ or Fisher's exact test as appropriate. Age groups: 1–5, 6–15, 16–25, 26–45, 46–65, > 65 years old.

## 4 Discussion and Conclusions

This study examined whether laboratory‐confirmed respiratory virus detections in hospital wards provide additional information beyond existing community and ED virological surveillance systems in Milan, Italy. Between 2024 and 2025, we observed differences in tested populations, overall positivity rates, and virus‐specific temporal patterns across settings. These findings support the view that surveillance limited to a single healthcare setting may incompletely capture the epidemiology of respiratory virus circulation.

The study included 8029 respiratory samples, mostly collected in the community, with smaller but relevant contributions from EDs and wards. This distribution reflects structural differences in surveillance coverage between primary care and hospital‐based settings. The ward population was older and more frequently male, whereas the community population included a higher proportion of younger individuals and females, consistent with known differences in healthcare‐seeking behavior and baseline risk [11, 12]. These complementary populations support integration of community‐ and hospital‐based laboratory surveillance rather than reliance on a single setting to describe respiratory virus circulation [2, 3].

The overall positivity rate for at least one virus was highest in the ED and community, and lower in wards. Because overall positivity was based on any virus among the targets tested for each sample, and because extended multiplex testing was not uniformly applied across all settings, these comparisons should not be interpreted as direct evidence of higher or lower underlying population incidence. Rather, they highlight the combined influence of viral circulation, healthcare‐seeking behavior, patient selection, and diagnostic testing practice. Similar contrasts in positivity rates across the community and hospital setting have been reported in Lyon, France, where community testing captured a large proportion of IV and SARS‐CoV‐2 cases, while hospital data provided additional insight into older and more vulnerable populations [2].

Overall, IAV was the most frequently detected virus across the full study period, followed by SARS‐CoV‐2. However, the ranking differed by setting: influenza viruses predominated in the community and ED settings, whereas PIV and SARS‐CoV‐2 were the most frequently detected among hospitalized patients. These differences likely reflect variations in case mix and clinical testing practices across healthcare settings. Hospitalized cohorts often include older patients, with multiple comorbidities, and more severe or atypical respiratory presentations [13]. From a public health perspective, incorporating ward‐based data may support more targeted prevention strategies and preparedness for high‐risk populations, but such data require careful interpretation because ward positivity alone does not measure community incidence or disease severity.

After low activity in mid‐2024, IAV was detected earlier in wards than in the community and ED, and the ward positivity peak also occurred first. This pattern suggests that ward‐based data may provide useful complementary signals for seasonal influenza activity among hospitalized patients. However, because the analysis of first detections and peaks was descriptive and some weekly denominators were small, these findings should not be interpreted as evidence that ward‐based surveillance is consistently earlier or superior to community or ED surveillance.

Age‐specific patterns further emphasized the importance of multi‐setting data. IAV was most frequently detected in ED individuals aged 1–5 years and > 65 years, whereas adults aged 46–65 years had the highest IAV positivity rate in the community. These differences likely reflect age‐related variation in exposure, susceptibility, disease severity, and healthcare‐seeking behavior [14]. This heterogeneous distribution across age groups and settings underscores the importance of age‐stratified surveillance data to inform targeted prevention strategies, including vaccination policies.

IBV showed a different profile, with higher positivity rates in the ED and community than in the ward setting and a concentration in younger age groups. In the 2024–2025 season, an Israeli study reported that IBV cases were predominantly observed in children and adolescents [15]. This aligns with our findings of comparatively higher IBV positivity rates in pediatric groups, most notably in 6–15‐year‐olds. Although IBV infection is sometimes perceived as less severe, hospital‐based studies in adults indicate that outcomes may be comparable to IAV, even if IBV patients tend to be younger in hospital cohorts [16]. These observations support considering IBV as an important driver of transmission in younger populations with meaningful clinical and public health implications.

RSV displayed a clear Autumn/Winter pattern, with variations in timing across settings. During the 2024–2025 Autumn/Winter, RSV was detected earlier in the community, while the positivity peak occurred first in community and ED and later in wards. In the youngest age group (1–5 years), RSV was detected more frequently in the ED, whereas no significant setting‐related differences were observed in other age groups. This pattern may reflect differences in healthcare‐seeking behavior, clinical presentation, and age‐specific testing practices, rather than true differences in viral circulation. In Lombardy, the introduction of a regional immunization campaign with a long‐acting monoclonal antibody from November 2024 may have modified RSV detection dynamics in pediatric populations. However, individual immunization data were unavailable and infants under 1 year were excluded; therefore, the impact of this intervention cannot be quantified in this study [17].

SARS‐CoV‐2 was detected throughout most of the study period, with relatively lower activity during the 2024–2025 Autumn/Winter period than during other phases, alongside substantial influenza activity. This apparent alternation in virus dominance is consistent with observations reported elsewhere in Europe. An integrated surveillance study from Geneva, Switzerland, combining primary care, hospital surveillance, and wastewater analysis, described the period from September 2024 to April 2025 as the first extended Winter season since the start of the COVID‐19 pandemic during which SARS‐CoV‐2 activity remained consistently lower than in previous seasons, while influenza virus activity was higher [18]. Together, these findings suggest a transition toward a post‐pandemic respiratory virus ecosystem in which SARS‐CoV‐2 circulation may increasingly resemble that of other seasonal respiratory viruses.

AdV, seasonal hCoV, and PIV circulated at lower levels and showed heterogeneous, age‐ and setting‐related patterns, consistent with previous reports indicating variable seasonality and geographic distribution [19, 20, 21]. These findings support the added value of multiplex surveillance beyond influenza viruses, RSV, and SARS‐CoV‐2 alone, especially for detecting shifts in less dominant but clinically relevant pathogens and for developing a more comprehensive picture of respiratory virus epidemiology [22].

This study has several limitations. First, the analysis was based on laboratory‐confirmed detections without associated clinical outcome data; therefore, the results describe viral detections, positivity, and temporal patterns, not disease severity, severe disease burden, or hospital pressure. Second, the populations tested in the community, ED, and ward settings differed in age distribution and testing indications, introducing selection bias that may have influenced both positivity rates and observed timing. Third, first detections and peak weeks were descriptive and may be unstable when weekly denominators are small.

Fourth, extended multiplex testing was applied uniformly in the community but only to subsets of ED and ward samples, limiting direct comparability of overall positivity and of AdV, hCoV, and PIV detections across settings. Fifth, the single metropolitan study setting may further limit the generalizability of the findings.

These limitations also define the scope of the public health implications. Viral circulation, severe disease burden, and hospital pressure are related but distinct concepts. Community surveillance is better suited to capturing broader respiratory virus circulation, ED data can identify acute healthcare demand among symptomatic patients, and ward data can characterize detections among hospitalized and older populations. However, without standardized clinical outcomes, hospitalization severity indicators, bed occupancy, length of stay, ICU admission, or mortality data, this study cannot quantify severe disease burden or hospital pressure.

In Lombardy, structured virological surveillance is already active in the community and ED, whereas hospital ward‐based surveillance is not systematically integrated into routine surveillance frameworks. The regional implementation of the MICRO‐BIO 2.0 platform represents a concrete opportunity to operationalize integrated surveillance at scale across all Lombardy healthcare providers [23]. By enabling standardized and automated collection of microbiological and virological data from hospital laboratories, this system could support routine inclusion of ward‐based data alongside existing community and ED systems. Wastewater‐based surveillance may provide additional complementary signals independent of healthcare‐seeking behavior and testing policies, with potential value for anticipating IAV and SARS‐CoV‐2 outbreaks [24].

In conclusion, ward‐based virological surveillance complements community and ED surveillance by contributing information on hospitalized and older populations. In this descriptive analysis, ward data did not consistently provide earlier signals than community or ED data, but they added context for selected pathogens and age groups. Integrating ward‐based laboratory data into existing surveillance frameworks through standardized testing algorithms and linkage with clinical outcome indicators could provide a more comprehensive and resilient picture of respiratory virus circulation and support evidence‐informed public health action during respiratory virus seasons.

## Author Contributions

**Alberto Rizzo:** conceptualization, data curation, formal analysis, visualization, writing – original draft, writing – review and editing. **Federica Salari:** data curation, writing – review and editing. **Cristina Galli:** data curation, writing – review and editing. **Manuel Maffeo:** data curation, writing – review and editing. **Cristina Paduraru:** data curation, writing – review and editing. **Simone Villa:** data curation, writing – review and editing. **Alberto Dolci:** writing – review and editing, supervision. **Elena Pariani:** conceptualization, writing – original draft, writing – review and editing. **Danilo Cereda:** conceptualization, writing – original draft, writing – review and editing. **Milan Respiratory Viruses Surveillance Working Group:** writing – review and editing, validation. All authors approved the final version of the manuscript.

## Funding

The authors have nothing to report.

## Ethics Statement

Ethical approval was waived because all human samples were collected as part of routine surveillance activities conducted by Health Authorities, and the analysis was conducted as part of public health practice. All data were anonymized and clinical samples were obtained during standard patient care.

## Conflicts of Interest

The authors declare no conflicts of interest.

## Supporting information


**Figure S1:** Weekly adenovirus (AdV), seasonal human coronaviruses (hCoV), and parainfluenza viruses 1–4 (PIV) cases among individuals tested at emergency department (ED), in hospital wards, or at general practitioners/pediatricians (community); X‐axis measures the week (from 1 January 2024, W1/2024, to 12 October 2025, W41/2025); Y‐axis measures the number of positive individuals (on the left). Weekly rate (%) of tests resulting positive at ED, wards and community; X‐axis measures the week; Y‐axis measures the positivity rate (No. of positive cases/No. of tested individuals) (on the right)

## Data Availability

The original data will be available upon reasonable request.
