# Supplementary material for: Integrated Hospital, Emergency Department, and Community Surveillance for Respiratory Viruses in Milan, Italy
Source: J Med Virol. 2026 Jul 21;98(8):e71073. doi: 10.1002/jmv.71073 (PMC13387720; doi:10.1002/jmv.71073)
Supplement: Supplementary file 1 — Figure S1: Weekly adenovirus (AdV), seasonal human coronaviruses (hCoV), and parainfluenza viruses 1–4 (PIV) cases among individuals tested at emergency department (ED), in hospital wards, or at general practitioners/pediatricians (community); X‐axis measures the week (from 1 January 2024, W1/2024, to 12 October 2025, W41/2025); Y‐axis measures the number of positive individuals (on the left). Weekly rate (%) of tests resulting positive at ED, wards and community; X‐axis measures the week; Y‐axis measures the positivity rate (No. of positive cases/No. of tested individuals) (on the right) [file JMV-98-e71073-s001.docx]

**Supplementary Figure 1.** Weekly adenovirus (AdV), seasonal human coronaviruses (hCoV), and parainfluenza viruses 1-4 (PIV) cases among individuals tested at emergency department (ED), in hospital wards, or at general practitioners/pediatricians (community); X-axis measures the week (from 1 January 2024, W1/2024, to 12 October 2025, W41/2025); Y-axis measures the number of positive individuals (on the left). Weekly rate (%) of tests resulting positive at ED, wards and community; X-axis measures the week; Y-axis measures the positivity rate (No. of positive cases/No. of tested individuals) (on the right).
